# CA-CAE: A deep learning-based multi-omics model for pan-cancer subtype classification and prognosis prediction

**DOI:** 10.1371/journal.pcbi.1014015

**Published:** 2026-02-20

**Authors:** Shumei Zhang, Yicheng Lu, Peixian Li, Junxuan Wu, Guohua Wang, Wen Yang

**Affiliations:** 1 College of Computer and Control Engineering, Northeast Forestry University, Harbin, China; 2 Department of Computer Science and Technology, Faculty of Computing, Harbin Institute of Technology, Harbin, China; 3 International Medical Center, Shenzhen University General Hospital, Shenzhen, China; University of Queensland, AUSTRALIA

## Abstract

In cancer research, identifying cancer subtypes and evaluating prognosis are crucial for personalized diagnosis and treatment of cancer. With the advancement of high-throughput sequencing technologies, multi-omics data has become essential for cancer classification and prognostic analysis. By integrating deep learning techniques, it is possible to more accurately identify cancer subtypes, providing a robust basis for personalized treatment of cancer patients. In this study, we propose a convolutional autoencoder prognostic model incorporating a channel attention mechanism (CA-CAE). The model utilizes multi-omics data to predict survival-associated cancer subtypes and identify prognostic genes. We applied CA-CAE to multiple cancer types, successfully identifying subtypes in 15 distinct cancer types and revealing significant survival differences among these subtypes. Moreover, compared to traditional statistical methods and other deep learning approaches, CA-CAE demonstrated superior performance in predicting survival outcomes.

## Author summary

Cancer is a highly complex disease, and predicting how a patient will respond to treatment or how their disease will progress is one of the biggest challenges in modern medicine. In this study, we developed a deep learning model called CA-CAE that combines multiple types of biological data—such as gene expression, DNA methylation, and microRNA levels—to identify cancer subtypes and predict patient outcomes more accurately. Unlike traditional models that rely on a single type of data or treat all features equally, our model uses a special attention mechanism to focus on the most important biological signals. We tested this approach on data from 15 types of cancer and found that it outperformed existing methods in identifying meaningful patient subgroups and predicting survival. These findings suggest that combining different layers of biological information can provide a more complete understanding of cancer and may help guide more personalized treatment strategies in the future. Our work highlights how artificial intelligence can be used to improve cancer care by making better use of the vast data already available.

## Introduction

In cancer research, determining cancer subtypes and prognosis is crucial. In previous studies, cancer prognosis analysis has mostly been conducted using single-omics datasets [1,2]. Although single-omics data can indeed identify some genes associated with prognosis [3], they only provide information at a specific omics level. Due to the complex multi-layered regulatory mechanisms of diseases like cancer, such a single-perspective approach may miss key information, failing to reveal the interactions and combined effects across different omics layers, thus limiting the predictive power of prognostic models [4]. With advances in technology and reduced costs, multi-omics datasets have gradually been used in cancer research [5]. Compared to single-omics, multi-omics emphasizes comprehensiveness and systematicity, helping to understand the functioning of biological systems holistically, thereby providing a more complete reflection of cellular characteristics [6,7]. However, since the magnitude of data values in different omics layers may vary significantly, integrating them without normalization can lead to data bias toward a particular omics type, making the integration and processing of multi-omics data very complex.

To address these issues, machine learning and deep learning methods have been used to integrate multi-omics data [8,9].Linear methods such as Principal Component Analysis (PCA) and Canonical Correlation Analysis (CCA) can identify correlated features across omics datasets but rely on linear assumptions and are insufficient for capturing nonlinear dependencies [10,11]. Matrix factorization approaches such as Non-negative Matrix Factorization (NMF) and Multi-Omics Factor Analysis (MOFA) can uncover shared latent structures, yet they are sensitive to noise and require manual tuning of the number of components, which limits scalability across diverse datasets. Similarity Network Fusion (SNF) integrates sample-level similarities but fails to learn complex hierarchical feature interactions. As multi-omics data continue to grow in size and complexity, these methods struggle to generalize and often depend on extensive feature engineering.

With the continuous development of deep learning, many deep learning-based multi-omics integration methods have been used in cancer research. Autoencoders (AEs), which include an encoder and a decoder, are a type of special Artificial Neural Network (ANN) model. They extract features by minimizing the reconstruction error in an unsupervised manner. The raw input data is first mapped to a low-dimensional representation space to obtain the most suitable features, and then the decoder maps the features in the low-dimensional space back to the input space. The loss between the original input to the encoder and the output of the decoder is used as a training error to optimize the model [12]. DeepProg is a collection of deep learning and machine learning models that uses autoencoders for multi-omics data to predict prognosis and cancer subtypes related to patient survival. However, autoencoders cannot account for multidimensional aspects during dimensionality reduction, ignoring correlations among biological features [13]. Convolutional Neural Networks (CNNs), as a representative deep learning algorithm [14], have promoted the development of various applications such as medical imaging [15]. Convolutional autoencoders (CAEs) [16] combine the advantages of CNNs and autoencoders. Li and Wang used convolutional autoencoders for scalable and accurate studies of spatial transcriptomics [17]. CAEs utilize convolutional filters to learn information from highly correlated features across multiple datasets, allowing them to identify features that are highly related to cancer prognosis.

Liu and Song proposed a convolutional autoencoder-based prognostic model (ProgCAE), which used CAEs to integrate multi-omics datasets for prognostic prediction [18]. However, ProgCAE directly applied convolution and autoencoder operations for feature extraction, assigning uniform weights to each feature channel without dynamically adjusting for the importance of different channels. This might lead to unnecessary weights being assigned to minor features, affecting the focus on key features. Although ProgCAE has advantages in integrating and processing multi-omics data, it has limited capacity for capturing nonlinear correlations among feature channels, which can result in suboptimal performance when handling highly nonlinear, multi-layered data.

Channel attention mechanisms are widely used in medical image segmentation, natural language processing, object detection, and many other fields, and their effectiveness has been well-established in various domains. Their core role is to dynamically adjust the weight of feature channels, allowing models to focus more precisely on key features and improve task accuracy. Tang and Luo developed a multi-view multi-channel attention graph convolutional network to predict potential associations between miRNAs and diseases [19]. G.Maheswari and S.Gopalakrishnan introduced a dynamic channel attention mechanism to enhance spatial feature extraction in medical image analysis, significantly improving diagnostic outcomes [20]. In multi-omics integration, the importance of different omics features can vary. The introduction of a channel attention mechanism can help the model more precisely focus on feature channels that significantly influence survival prognosis, enhancing the predictive performance and stability of the model.

Therefore, this study developed a pan-cancer prognostic model based on a Convolutional Autoencoder with Channel Attention (CA-CAE). The model is used to integrate multi-omics data for cancer classification and prognosis prediction. CA-CAE utilizes the following omics datasets as inputs: DNA methylation, RNA-seq, and miRNA-seq. We applied CA-CAE to 15 types of cancer, and our model demonstrated better prediction accuracy compared to many other published methods.

## Results

### Identification of survival-related subgroups in 15 cancer datasets

To determine survival-related subgroups within the datasets, we obtained 15 cancer multi-omics datasets, including mRNA-seq, miRNA-seq, and DNA methylation data, from the TCGA cancer database. We preprocessed these data using the standard procedure of data normalization and feature selection. For each dataset, we conducted dimensionality reduction and feature transformation to identify potential survival features, which were then used for clustering to identify survival-related subgroups.

**Fig 1:**
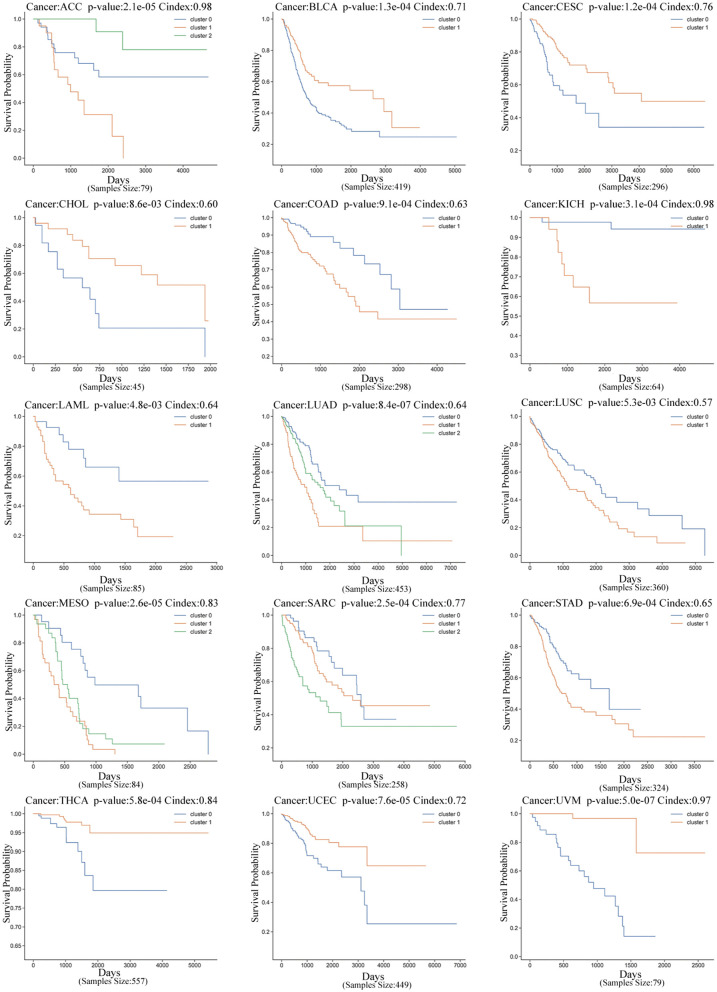
Kaplan-Meier survival curves for cancer subgroups identified using CA-CAE across 15 multi-omics cancer datasets. Each panel represents one cancer type, with survival-related subgroups identified through K-means clustering.

The selected features were first filtered using LASSO regression to select features with non-zero Lasso coefficients. These selected features were then used in a univariate Cox proportional hazards model (Cox-PH) to further screen features associated with survival (P< 0.05, based on the log-rank test). The features that passed this selection process were used to build a new feature matrix. Next, we used these features for K-means clustering, and the optimal number of clusters was determined by analyzing the clustering results. We found that 8 cancer types had an optimal number of clusters equal to 2, while 4 cancer types had an optimal number of clusters equal to 3. The cluster numbers for each cancer type are summarized in S1 Table, and **Fig 1** shows the survival analysis results for CA-CAE across different cancer types. Detailed cluster numbers are further summarized in **Table 1**.

**Table 1:** Summary of number of clusters.

Cancer	Number of clusters
ACC	3
BLCA	2
CESC	2
CHOL	2
COAD	2
KICH	2
LAML	2
LUAD	3
LUSC	2
MESO	3
SARC	3
STAD	2
THCA	2
UCEC	2
UVM	2

### Comparison with other deep learning models

To demonstrate the advantage of our model, we compared it with three other deep learning models and one traditional matrix factorization baseline (NMF). In **Table 2**, the CA-CAE model achieved lower P-values in 9 cancer types (e.g., LUAD, UVM, ACC), demonstrating better performance. These P-values are obtained from log-rank tests, which evaluate the statistical significance of survival differences between the subtypes identified by each model. Lower P-values indicate that the survival curves of the predicted subtypes are more significantly separated, suggesting stronger prognostic discrimination ability. In LUAD, the P-value for CA-CAE is 8.4e-07, which is significantly smaller compared to other models (e.g., ProgCAE with P-value of3.5e-03). This indicates that CA-CAE achieves more robust statistical significance in predicting survival for cancer types, particularly during repeated cross-validation training.

**Table 2:** Summary of P-values for different models (Log-rank test results).

Cancer	CA-CAE	ProgCAE	DeepProg	PCA	NMF
ACC	2.1e-05	1.2e-03	1.6e-05	5.9e-06	**6.4e-07**
BLCA	**1.3e-04**	3.7e-02	6.6e-03	5.4e-04	1.6e-01
CESC	**1.2e-04**	8.4e-01	1.3e-02	4.2e-03	9.4e-01
CHOL	**8.6e-03**	1.0e-01	5.3e-01	2.0e-02	2.9e-01
COAD	**9.1e-04**	1.9e-02	1.7e-03	3.4e-03	4.3e-01
KICH	**3.1e-04**	1.2e-03	2.3e-01	1.7e-01	2.2e-01
LAML	4.8e-03	1.4e-02	**1.1e-03**	3.7e-02	6.3e-01
LUAD	**8.4e-07**	3.5e-03	2.5e-03	1.7e-02	1.3e-01
LUSC	5.3e-03	2.2e-03	2.5e-02	**4.5e-03**	3.5e-01
MESO	2.6e-05	2.3e-06	2.6e-04	**5.6e-07**	2.3e-02
SARC	**2.5e-04**	4.5e-03	3.0e-04	2.1e-02	2.7e-03
STAD	**6.9e-04**	1.5e-03	2.9e-02	8.1e-02	3.7e-02
THCA	**5.8e-04**	1.3e-02	3.0e-01	1.2e-01	2.1e-01
UCEC	7.6e-05	**1.5e-08**	8.3e-05	2.1e-04	1.5e-04
UVM	**5.0e-07**	1.3e-06	1.9e-05	8.4e-06	2.1e-02

In **Table 3**, the CA-CAE model demonstrates robust predictive power, outperforming other models in 10 cancer types. The C-index for CA-CAE is notably higher in comparison with the other three models. The C-index measures the ability of the model to correctly predict patient outcomes, with a higher value indicating better performance(see Model Evaluation Metrics- Cox-PH Concordance Index for a detailed description of this metric). Compared to CA-CAE, ProgCAE and PCA do not perform as well in many cases.In contrast, NMF shows the lowest average C-index among all models, again emphasizing that deep nonlinear architectures such as CA-CAE and ProgCAE provide more informative representations for survival prediction than traditional matrix factorization methods.

**Table 3:** C-index (Concordance index) comparison among different prognostic models.

Cancer	CA-CAE	ProgCAE	DeepProg	PCA	NMF
ACC	**0.98**	0.96	0.74	0.84	0.79
BLCA	**0.71**	0.70	0.54	0.70	0.55
CESC	**0.76**	0.71	0.63	0.75	0.54
CHOL	**0.60**	0.60	0.54	0.59	0.59
COAD	0.63	0.53	0.56	**0.73**	0.53
KICH	**0.98**	0.92	0.62	0.95	0.64
LAML	0.64	0.71	0.62	**0.79**	0.54
LUAD	0.64	**0.67**	0.63	0.66	0.57
LUSC	0.57	0.58	0.57	**0.62**	0.55
MESO	**0.83**	0.81	0.61	0.78	0.60
SARC	**0.77**	0.75	0.62	0.76	0.64
STAD	**0.65**	0.62	0.55	0.63	0.58
THCA	0.84	0.77	0.54	**0.91**	0.56
UCEC	**0.72**	0.69	0.61	0.71	0.61
UVM	**0.97**	0.90	0.77	0.84	0.72

Collectively, these results demonstrate that CA-CAE offers more reliable and biologically meaningful stratification of cancer patients across multiple datasets.

**Fig 2:**
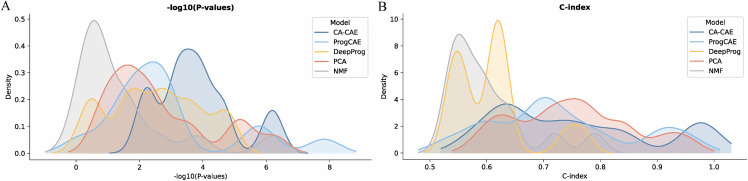
Comparison of model performance in terms of -log10(P-values) and C-index for five models across 15 cancer types. **(A)** Distribution of -log10(P-values) for CA-CAE, ProgCAE, DeepProg, PCA and NMF. **(B)** Distribution of C-index values for the same five models.

To further compare the performance of CA-CAE with other models, we analyzed the distribution of -log10(P-values) and C-index values across the 15 cancer types. **Fig 2A** demonstrates that CA-CAE consistently achieves higher -log10(P-values), indicating greater statistical significance in identifying survival-associated features. **Fig 2B** further highlights the superior predictive accuracy of CA-CAE, as evidenced by higher C-index values compared to ProgCAE, DeepProg, and PCA.

Although the CA-CAE model does not achieve the highest score for every individual cancer type, an integrated analysis of both P-value and C-index shows that CA-CAE still delivers the most reliable performance. It achieves high predictive accuracy across different models and effectively stratifies patients, making it a strong candidate model for robust cancer subtype classification.

Furthermore, to assess the reliability of the survival prediction results, 95% confidence intervals (CIs) for the C-index were computed using bootstrap resampling.As summarized in **Table 4**, the confidence intervals across all cancer types are narrow, indicating stable and reproducible model performance, suggesting high robustness of the CA-CAE model across datasets.

**Table 4:** Summary of 95% confidence intervals for the C-index across 15 cancer types.

Cancer	C-index	95% CI Lower	95% CI Upper
ACC	0.980	0.973	0.987
BLCA	0.709	0.691	0.727
CESC	0.762	0.744	0.781
CHOL	0.603	0.569	0.634
COAD	0.631	0.605	0.659
KICH	0.982	0.970	0.990
LAML	0.647	0.615	0.672
LUAD	0.643	0.619	0.662
LUSC	0.576	0.544	0.603
MESO	0.833	0.812	0.851
SARC	0.775	0.751	0.789
STAD	0.657	0.627	0.676
THCA	0.845	0.823	0.861
UCEC	0.724	0.703	0.743
UVM	0.972	0.959	0.983

### Ablation study

**Fig 3:**
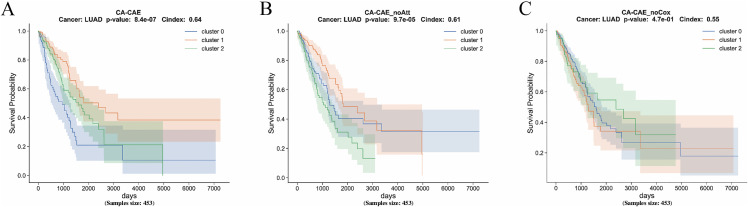
Ablation study of CA-CAE components on LUAD. Kaplan–Meier survival curves of LUAD patients illustrating the impact of different modules on model performance:(A) The full CA-CAE model achieved the highest discriminative power. **(B)** Removing the channel attention mechanism (CA-CAE_noAtt) led to decreased stratification performance. **(C)** Excluding the Cox-based prognostic filtering (CA-CAE_noCox) further reduced discrimination.

To assess the contribution of each component in the CA-CAE framework, ablation experiments were conducted on the LUAD dataset. As shown in **Fig 3**, removing either the channel attention mechanism or the Cox-based prognostic filtering notably reduced the model’s discriminative ability. Specifically, the model without the Cox module (CA-CAE_noCox) exhibited a much higher p-value (p = 4.7 e-01) and a lower C-index (0.55), while removing the attention mechanism (CA-CAE_noAtt) led to moderate degradation (p = 9.7e-05, C-index = 0.61). The full CA-CAE model achieved the best performance (p = 8.4e-07, C-index = 0.64), confirming the effectiveness and necessity of these components in enhancing feature selection and prognostic prediction.

### Construction and prediction of classifiers

Although the CA-CAE model primarily performs unsupervised clustering to identify potential cancer subtypes, we further employed a supervised classification step to validate the robustness of these clusters. Specifically, the K-means algorithm first discovers subtype groupings without using label information, while the subsequent SVM classifier is trained using the obtained cluster labels as pseudo-labels. This two-stage design allows the model to both uncover intrinsic subtype structures and assess their reproducibility through supervised prediction.

**Fig 4:**
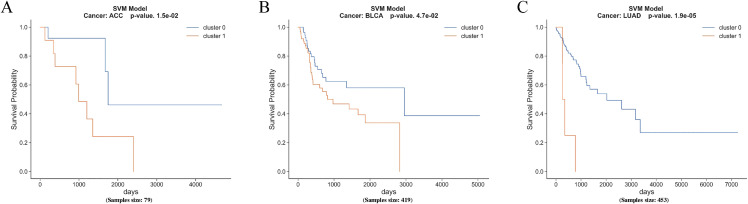
Kaplan-Meier survival curves for SVM model predictions in ACC, BLCA, and LUAD datasets.

In this study, we constructed classifiers based on multi-omics data to validate the clustering results obtained from unsupervised learning. We utilized support vector machine (SVM) classifiers for ACC, BLCA, and LUAD data to predict the classification labels obtained from clustering. As shown in **Fig 4**. Following normalization, we selected the top 50 features with the highest variance from each omics dataset and combined them into a new feature matrix. The dataset was then split into a 70/30 ratio for training and testing. The training set was used to train the SVM model, while the testing set evaluated the model’s prediction capability.

To select the optimal hyperparameters for the SVM classifier, we performed a grid search with five-fold cross-validation using the scikit-learn library. All predictions were subsequently evaluated using Kaplan-Meier survival analysis, and the significance of each classification was determined using a log-rank test with a significance threshold of 0.05.

### Differences between multi-omics integration and single omics analysis

This study investigates the effectiveness of integrating multiple omics features in predicting cancer patient survival outcomes, compared to using single omics analysis. Specifically, we analyzed LUAD patients using mRNA data to identify significant features. These features were extracted and analyzed through univariate Cox regression to examine their impact on survival outcomes. Additionally, we applied the log-rank test to validate the differences between the subgroups.

**Fig 5:**
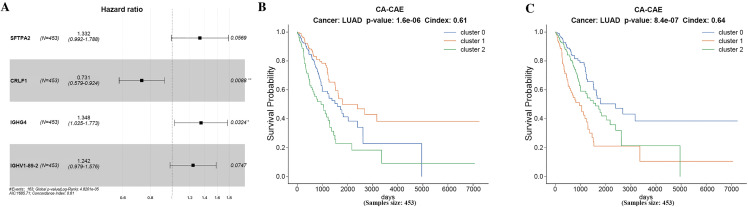
Comparison of single-omics and multi-omics approaches in predicting survival outcomes for LUAD patients. **(A)** Hazard ratio analysis of significant mRNA features identified through univariate Cox regression in single-omics analysis. **(B)** Kaplan-Meier survival curve based on mRNA single-omics features for LUAD patients. **(C)** Kaplan-Meier survival curve based on integrated multi-omics features (mRNA and DNA methylation) for LUAD patients.

The integrative multi-omics analysis demonstrated significantly enhanced prognostic accuracy in survival prediction compared to single-omics approaches. In particular, the combined Cox regression analysis showed significant differences, highlighting that different omics provide complementary information. **Fig 5A** demonstrates that CRLF1 and IGHG4 were among the top features identified for LUAD patients, showing significant p-values and strong predictive capabilities.

For patients with the same clinical stage, integrating multiple omics features (such as mRNA and methylation) for survival prediction provided a more stable outcome compared to using single omics. As illustrated in **Fig 5B** and **5C**, the Kaplan–Meier survival curves demonstrate that the integrated multi-omics approach provides significantly better prognostic performance compared to single-omics methods, with p-values less than 0.05, indicating the advantage of the integrated approach. Although both approaches demonstrate some predictive value, the p-value from the multi-omics model is smaller, and the C-index also shows that integrated features lead to better prediction.

In conclusion, this study demonstrates that the integration of multiple omics features provides more robust survival prediction for cancer patients. Our results suggest that integrated analysis is superior in capturing the underlying biological variability compared to single omics analysis, especially in distinguishing patient subgroups and predicting long-term survival outcomes.

In this study, we extracted and analyzed the top 20 ranked genes across three omics datasets for LUAD cancer. As shown in **Table 5**. In the mRNA dataset, SFTPA1 and SFTPA2 have been demonstrated to increase cancer risk in mutation carriers [21]. GPX2 expression is closely associated with prognosis in lung adenocarcinoma patients [22], and CEACAM5 is a reliable marker for the pre-screening of lung adenocarcinoma tumor cells via RT-PCR [23]. MSLN expression is identified as a marker of tumor invasiveness and a potential therapeutic target [24]. S100P has been shown to be useful for prognosis analysis by combining features of lung adenocarcinoma heterogeneity and microenvironment at the single-cell level [25]. High levels of AKR1C1-STAT3are strongly associated with poor prognosis in NSCLC patients [26]. A negative correlation between plasma proteins SFTPB and KDELC2in LUAD was observed using bidirectional Mendelian randomization (MR) and colocalization analysis [27]. PIGR is an independent prognostic biomarker for EGFR-TKI resistance and tumor immune cell infiltration in LUAD [28]. TFF3effectively promotes LUAD progression, and targeting TFF3 with novel small molecule inhibitors, alone or combined with traditional MEK1/2 inhibitors, may improve LUAD prognosis [29]. MUC5B-AS1promotes cell migration and invasion in LUAD by forming an RNA-RNA duplex with MUC5B, whose high expression is significantly associated with poor outcomes in LUAD [30]. CPS1, used in combination with other chemotherapeutic drugs, is a promising therapeutic target and a prognostic biomarker for personalized treatment of LUAD [31]. CTSE is overexpressed and hypomethylated in LUAD compared to normal lung tissue [32]. TFPI-2gene methylation is an independent factor for poor prognosis in NSCLC patients [33]. CXCL14 is a potential diagnostic biomarker for LUAD with micropapillary patterns [34]. Two common risk genes (SFTPD and HLA-DRA) at different stages are identified as prognostic markers for LUAD [35]. The number of tumor-infiltrating lymphocytes (TILs) within tumor nests is an independent prognostic marker for patients with LUAD brain metastasis. Transcriptomic analysis showed that three surfactant metabolism-related genes (SFTPA1, SFTPB, and NAPSA) are closely correlated with the number of TILs [36]. Upregulation of genesAKR1C1/C2, TM4SF1, andNR0B1 in the side population (SP) of LUAD A549 cells might be targets for poor prognosis in anticancer therapies [37].

**Table 5:** Summary of top 20 ranked genes across 3 omics datasets.

mRNA	Meth	miRNA
SFTPA1	FHL1	hsa-mir-205
SFTPA2	G6PD	hsa-mir-9–2
GPX2	DOCK11	hsa-mir-34c
CEACAM5	XK	hsa-mir-192
MSLN	UXT	hsa-mir-194-2
S100P	LOC391322	hsa-mir-194-1
AKR1C2	MTMR1	hsa-mir-210
SFTPB	AFF2	hsa-mir-375
PIGR	BEX4	hsa-mir-3607
TFF3	MSL3	hsa-mir-486-2
MUC5B	RIMS2	hsa-mir-486-1
CPS1	PDK3	hsa-mir-224
CTSE	BCAP31	hsa-mir-451a
TFPI2	PRKCB	hsa-mir-203a
IGHD	FAM120C	hsa-mir-3065
CXCL14	CCDC160	hsa-mir-338
CRLF1	WDR45	hsa-mir-149
SFTPD	MOSPD1	hsa-mir-144
NAPSA	NR0B1	hsa-mir-10b
AKR1C1	NKRF	hsa-mir-582

In summary, we conducted an in-depth analysis of the top 20 ranked genes across three omics datasets for LUAD, identifying several potential biomarkers and therapeutic targets of significant clinical relevance. These genes not only reveal the molecular mechanisms underlying LUAD but also provide new perspectives for the study of prognostic molecular markers. These findings establish a crucial foundation for exploring molecular mechanisms in pan-cancer studies and developing precision medicine strategies, emphasizing the importance of systematic analysis within a multi-omics data context. In the future, further cross-cancer big data integration and functional validation experiments could identify more common and specific biomarkers, opening new avenues for precise diagnosis and personalized treatment of cancer.

### Functional and pathway enrichment analysis

In this study, we performed functional and pathway enrichment analysis on survival-related genes extracted from the LUAD dataset using the CA-CAE model. The aim was to explore the potential role of these genes in immune response and cancer progression. We conducted Gene Ontology (GO) and KEGG pathway enrichment analyses for the survival-related genes extracted from LUAD.

**Fig 6:**
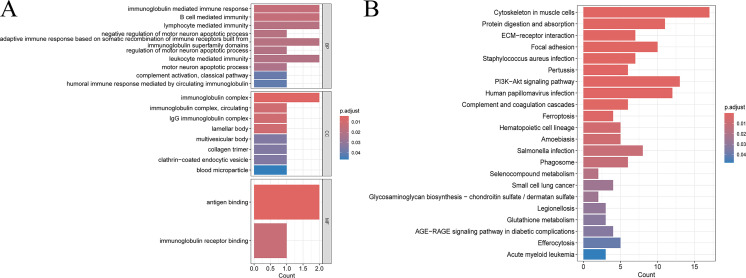
Functional and pathway enrichment analyses of survival-related genes identified from the LUAD dataset. **(A)** Gene Ontology (GO) enrichment analysis results. (B) KEGG pathway enrichment analysis results.

**Fig 6A** presents the results of the GO enrichment analysis, which revealed that the selected genes are highly enriched in immune response–related terms.

Specifically, in the Biological Process (BP) category, genes were significantly enriched in immune system activation, regulation of immune response, and antigen presentation. These findings suggest that the selected genes may play a critical role in modulating the tumor microenvironment and enhancing the immune system’s antitumor response.

In the Cellular Component (CC) category, the genes were primarily enriched in components related to the extracellular matrix, suggesting their involvement in tumor invasion and metastasis. Finally, in the Molecular Function (MF) category, these genes were enriched in immune receptor binding and antigen binding activities, indicating their potential role in recognizing and neutralizing tumor cells.

**Fig 6B** presents the results of KEGG pathway enrichment analysis. The identified genes are significantly enriched in pathways related to cell adhesion, extracellular matrix (ECM) interactions, and integrin signaling, suggesting their potential roles in tumor invasion, adhesion, and migration. Moreover, enrichment in the PI3K-Akt signaling pathway indicates a potential involvement in regulating cell proliferation and survival, further implicating these genes in tumor growth and progression.

Additionally, the genes were also enriched in immune response pathways, including autoimmune diseases, asthma, and viral infections, indicating that these genes may play a role in modulating the immune response to cancer. Moreover, complement and coagulation cascade analysis suggested that these genes could have potential roles in enhancing or inhibiting immune-mediated inflammation in the tumor microenvironment.

### Associations between cancer subtypes and clinical features

This study aims to evaluate the associations between cancer subtypes and clinical features to assess their potential value in indicating personalized treatment strategies during cancer progression. Specifically, we analyzed the relationships between clinical features (Stage, T, N, M) and subtypes for BLCA, COAD, and LUAD. The P-values reported in this section are derived from Chi-square tests, which examine whether there are statistically significant associations between categorical clinical variables and the predicted cancer subtypes. Lower P-values indicate stronger dependence between subtypes and clinical features, suggesting that the identified molecular subtypes may reflect distinct clinical characteristics. Chi-square tests were conducted to evaluate the associations and to determine if there were significant correlations between subtypes and clinical features.

**Table 6** shows that in BLCA, the Stage variable is significantly associated with subtypes (p = 0.0005), indicating a strong relationship that could inform personalized treatment strategies. Similarly, the T variable (p = 0.0004) is also significantly associated, while N (p = 0.2336) and M (p = 0.3716) show no significant associations.

**Table 6:** Associations between clinical features and cancer subtypes (Chi-square test results).

BLCA	Variable	Chi-square Statistic	P-value	Significance
	Stage	17.69	0.0005	Significant
	T	17.81	0.0004	Significant
	N	4.27	0.2336	Not Significant
	M	0.79	0.3716	Not Significant
COAD	Variable	Chi-square Statistic	P-value	Significance
	Stage	19.11	0.0002	Significant
	T	6.48	0.09	Not Significant
	N	16.95	0.0002	Significant
	M	11.70	0.0006	Significant
LUAD	Variable	Chi-square Statistic	P-value	Significance
	Stage	14.31	0.0263	Significant
	T	8.56	0.1998	Not Significant
	N	12.70	0.0128	Significant
	M	2.59	0.2736	Not Significant

For COAD, the variables Stage, N, and M were all significantly associated with subtypes (P< 0.05), whereas the T variable did not show a significant association (p = 0.09). These results suggest that staging, lymph node involvement, and distant metastasis may serve as important markers for distinguishing between different subtypes in COAD patients, providing a foundation for personalized treatment approaches.

For LUAD, the Stage variable (p = 0.0263) and the N variable (p = 0.0128) were significantly associated with subtypes, whereas T (p = 0.1998) and M (p = 0.2736) were not significantly associated. These findings suggest that staging and lymph node involvement may play key roles in identifying LUAD subtypes, offering further insights into personalized treatment strategies for these patients.

### Association of CA-CAE subtypes with WGD and NMF

To further assess the biological relevance of the CA-CAE–derived cancer subtypes, we examined their associations with whole-genome doubling (WGD), a key hallmark of cancer evolution [38,39]. For each cancer type, we compared the distribution of WGD-positive and WGD-negative samples across subtypes using χ² tests and quantified effect sizes by Cramér’s V. False discovery rate (FDR) correction was applied across cancers (α = 0.05).

As shown in **Table 7**, significant associations between WGD status and CA-CAE subtypes were observed in COAD (FDR = 8.8 × 10 ⁻ ⁷, Cramér’s V = 0.33), LUAD (FDR = 4.1 × 10 ⁻ ⁵, Cramér’s V = 0.23), and SARC (FDR = 2.0 × 10 ⁻ ⁶, Cramér’s V = 0.35). These cancers exhibited distinct subtype-specific WGD enrichment patterns, suggesting that CA-CAE successfully captures genomic instability signals that are biologically and clinically meaningful. In other cancer types, no significant associations were found (FDR > 0.05), although COAD and LUAD demonstrated particularly strong subtype–WGD coupling consistent with prior observations of chromosomal instability–driven heterogeneity.

**Table 7:** Associations between CA-CAE subtypes and WGD status across cancers.

Cancer	k	n_samples	WGD rate	χ²	p-value	FDR-adjusted p	Cramér’s V	Significance
COAD	2	276	0.41	29.27	6.3e-08	8.8e-07	0.33	significant
LUAD	3	433	0.58	23.31	8.7e-06	4.1e-05	0.23	significant
SARC	3	244	0.46	30.09	2.9e-07	2.0e-06	0.35	significant
ACC	3	76	0.50	8.26	1.6e-02	5.6e-02	0.33	borderline

To further validate the capability of the CA-CAE model in identifying biologically meaningful cancer subtypes [40], we compared its clustering outcomes with those obtained using the NMF-based approach previously applied to lung cancer. Instead of replicating the specific subtypes identified by NMF, this comparison aimed to evaluate whether CA-CAE could achieve consistent and biologically relevant subtype stratification.

Across 15 cancer types, the agreement between CA-CAE and NMF results—quantified by the Adjusted Rand Index (ARI) and Normalized Mutual Information (NMI)—ranged from low to moderate. As shown in **Table 8,** SARC, LUAD, and CESC exhibited higher consistency, suggesting that CA-CAE can recognize subtype structures comparable to those identified by NMF while uncovering additional nonlinear patterns.

**Table 8:** Comparison of CA-CAE and NMF clustering consistency across representative cancer types.

Cancer Type	ARI	NMI	Comment
SARC	0.490	0.549	Strong consistency
LUAD	0.306	0.336	Moderate-to-high consistency
CESC	0.345	0.298	Moderate consistency

Overall, these findings demonstrate that the CA-CAE model can reliably identify stable and biologically interpretable subtypes across diverse multi-omics datasets.

### Generalization to proteogenomic data (CPTAC datasets)

**Fig 7:**
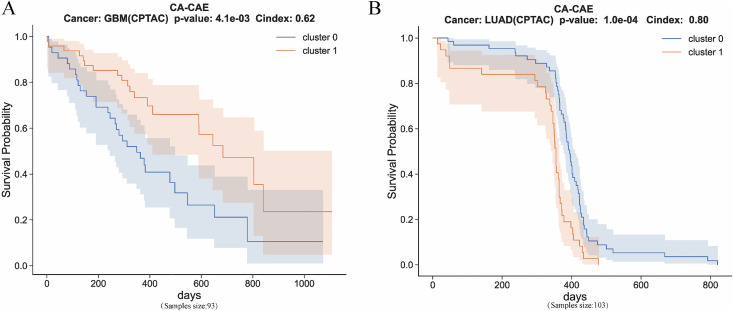
Kaplan–Meier survival analysis for GBM and LUAD patients from the CPTAC cohort using the CA-CAE model. **(A)** Cancer: GBM (CPTAC). **(B)** Cancer: LUAD (CPTAC).

To further validate the generalizability of the CA-CAE model, we applied it to multi-omics datasets from the Clinical Proteomic Tumor Analysis Consortium (CPTAC) [41,40] (**Fig 7**). All datasets were obtained from the Proteomic Data Commons platform (https://pdc.cancer.gov/pdc/browse). Specifically, we selected two cancer cohorts, GBM (glioblastoma multiforme) and LUAD (lung adenocarcinoma), which contain proteomic, transcriptomic, and patient survival information.

During preprocessing, we first matched samples across omics layers and retained only patients with both proteomic and transcriptomic data to ensure input consistency. The curated datasets were then fed into the CA-CAE model for training and validation to assess its prognostic prediction capability under integrated multi-omics conditions.

The results showed that in the GBM cohort, the model achieved significant survival stratification (p = 4.1e-03, C-index = 0.62), while in the LUAD cohort, the separation was even more pronounced (p = 1.0e-04, C-index = 0.80). These findings demonstrate that the CA-CAE model can effectively capture prognostic signals embedded within proteogenomic data and exhibits strong robustness and adaptability across omics modalities. Moreover, this cross-domain validation highlights the model’s potential for uncovering the synergistic effects between protein expression and transcriptional regulation, paving the way for its broader application in clinical multi-omics research.

## Discussion

This study proposed CA-CAE, a channel-attention-based convolutional autoencoder framework for multi-omics cancer subtype identification and prognosis prediction. By introducing a nonlinear feature extraction process coupled with an attention mechanism, the model was able to effectively integrate heterogeneous omics data and emphasize biologically informative features across multiple molecular layers. The experimental results on 15 TCGA cancer datasets demonstrated that CA-CAE achieves consistent and statistically significant separation of survival curves, validating its ability to uncover prognostically relevant subtypes in diverse cancer types.

Compared with existing deep learning and traditional clustering approaches, CA-CAE provides both improved predictive accuracy and greater robustness. The model consistently achieved lower log-rank P-values and higher C-index values across most cancer types, indicating stronger discriminative power for survival prediction. Moreover, the bootstrap-based confidence intervals of the C-index were narrow, confirming the model’s stability. These results suggest that CA-CAE effectively captures nonlinear dependencies and cross-omics interactions that are often overlooked by conventional dimensionality-reduction or matrix-factorization methods.

To further validate the reliability of the identified subtypes, we compared CA-CAE with the previously reported NMF-based clustering approach in lung cancer. The comparison, designed to assess the biological relevance of subtype separations rather than to replicate prior subtype labels, revealed moderate-to-high consistency between the two models. Cancers such as SARC, LUAD, and CESC exhibited the strongest agreement, with NMI values exceeding 0.3. This indicates that CA-CAE can recognize subtype structures consistent with those identified by NMF while uncovering additional nonlinear biological patterns. Such cross-method consistency provides strong evidence that the CA-CAE model identifies stable, reproducible, and biologically interpretable subtypes, thus enhancing confidence in its generalization ability across different omics contexts.

The proposed CA-CAE framework not only improves clustering performance but also demonstrates biological interpretability. The subtypes discovered in cancers such as LUAD and SARC were found to be significantly associated with whole-genome doubling and clinical features, confirming that the model captures genuine molecular heterogeneity rather than statistical artifacts. The channel attention mechanism contributes to this interpretability by adaptively weighting omics features according to their relevance, allowing the model to highlight expression, methylation, or miRNA patterns that are most critical for prognosis.

Overall, CA-CAE represents a significant advancement in deep learning-based multi-omics integration for cancer research. Its architecture combines nonlinear representation learning, attention-guided feature refinement, and Cox-based prognostic filtering, resulting in a model that is both accurate and biologically grounded. Importantly, the framework is flexible and can be extended to other large-scale proteogenomic or single-cell multi-omics datasets, highlighting its potential for broader translational applications in precision oncology.

Nevertheless, several limitations remain. The interpretability of deep latent representations is still constrained compared with traditional linear methods, and the current model relies on pre-selected omics features, which may introduce bias. Future work could incorporate explainable AI techniques, such as SHAP or integrated gradient analysis, to better interpret the contributions of individual genes. In addition, external validation using independent clinical cohorts or proteogenomic datasets would further strengthen the model’s clinical relevance. Despite these challenges, the present findings confirm that CA-CAE achieves accurate, stable, and biologically meaningful cancer subtype identification, providing a powerful tool for integrative cancer genomics and precision medicine research.

## Materials and methods

### Data collection and preprocessing

Fifteen multi-omics datasets for cancers were downloaded from the UCSC Xena genomic data-sharing platform (GDC) hub (https://gdc.xenahubs.net), including RNA-seq, DNA methylation, and miRNA-seq data. Additionally, survival follow-up data and phenotypic information for each cancer type were also collected. The summary of sample counts for each dataset is shown in **Table 9**.

**Table 9:** Summary of datasets.

Cancer	Number of common samples
ACC	79
BLCA	419
CESC	296
CHOL	45
COAD	298
KICH	64
LAML	85
LUAD	453
LUSC	360
MESO	84
SARC	258
STAD	324
THCA	557
UCEC	449
UVM	79

Initially, biological features with missing values in the RNA-seq, DNA methylation, and miRNA-seq data were removed. Next, undefined biological features in each of the three omics datasets were excluded, followed by removing samples with missing survival data.

For each type of cancer, we first filtered out the samples in each omics dataset to ensure consistency with the survival data, guaranteeing that subsequent analyses were conducted on the same cohort of samples. To reduce dimensionality and retain the most informative features, we selected the top 3,000 RNA, 100 miRNA, and 1,000 DNA methylation features based on their variance across samples.

These thresholds were chosen after preliminary experiments showing that including more features (e.g., 5000 RNA, 200 miRNA) led to marginal improvement in performance (<2% change in C-index) but substantially increased computational cost and model complexity.Conversely, smaller feature sets (e.g., 1000RNA, 50 miRNA) reduced the model’s discriminative power.

To assess robustness, we conducted a sensitivity analysis by varying the feature counts within ±10% of the selected values.The overall performance (in terms of P-value and C-index) remained stable, indicating that the CA-CAE framework is not overly sensitive to the exact feature selection thresholds.The detailed results of the sensitivity analysis regarding feature selection thresholds are presented in S2 Table.

All data preprocessing and analyses were completed prior to April 4, 2025.

### Normalization

First, Min-Max normalization [42] was applied to linearly transform the values of the dataset into a fixed range, scaling the data such that the minimum value becomes 0 and the maximum value becomes 1, while other values are linearly transformed within this range. The Min-Max normalization formula is as follows:


$$\begin{array}{c} x^{\prime }=\frac{x-x_{\text{min}}}{x_{\text{max}}-x_{\text{min}}} \end{array}$$ (1)

Where $x^{\prime }$ represents the normalized value, x is the original data point, $x_{min}$ is the minimum value, $x_{max}$ is the maximum value.

Next, Pearson correlation coefficient [43] was utilized for data processing. Pearson’s correlation coefficient is used to assess the linear correlation between two variables. By calculating the correlations between columns, those with strong correlations can be identified. Pearson correlation analysis was applied to evaluate pairwise relationships among features, and highly correlated variables were filtered to avoid redundancy.

We calculated the geometric mean of each row in the correlation matrix, and based on the magnitude of these values, features were reordered accordingly.


$$\begin{array}{c} \rho =\sqrt[{p}]{\left|\rho_{\text{1}}\right|\cdot \left|\rho_{\text{2}}\right|\cdot \cdots \cdot \left|\rho_{n}\right|} \end{array}$$ (2)

where ρ represents the geometric mean of the cumulative correlation coefficients. $\rho_{\text{1}},\rho_{\text{2}},\ldots ,\rho_{n}$ denote the absolute values of all correlation coefficients in a specific row of the correlation matrix.

### Overview of CA-CAE

CA-CAE is used for patient stratification and prognosis evaluation based on three types of omics data (DNA methylation, mRNA-seq, and miRNA-seq). The first stage of CA-CAE consists of dimensionality reduction and feature transformation, utilizing Pearson correlation normalization and a convolutional autoencoder integrated with an attention mechanism for dimensionality reduction. Each of the three omics datasets is modeled by a convolutional autoencoder with an attention mechanism, enabling flexibility and scalability for heterogeneous data types.

**Fig 8:**
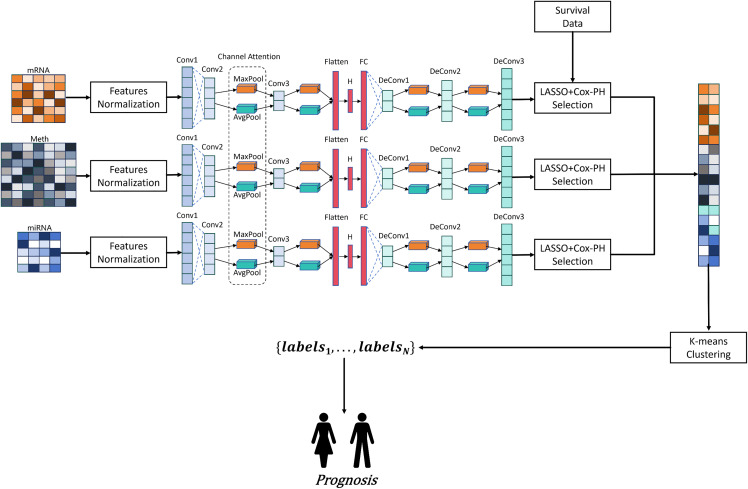
Overview of CA-CAE: The model includes feature normalization, dimensionality reduction, feature selection, and survival analysis for three types of omics data (DNA methylation, mRNA-seq, and miRNA-seq). Each omics dataset is modeled with a convolutional autoencoder (CAE) combined with an attention mechanism to improve flexibility and scalability for heterogeneous data types.

As shown in **Fig 8**, the CA-CAE framework includes three main steps: dimensionality reduction, feature selection, and survival analysis. After dimensionality reduction, the transformed features are first subjected to LASSO regression analysis to select features with non-zero Lasso coefficients. These features are then fitted using a univariate Cox proportional hazards model (Cox-PH) to further identify survival-associated features. Finally, the survival features from each omics dataset are combined for subsequent survival analysis and cancer subtype selection.

### Channel attention module

The effectiveness of attention mechanisms has been widely demonstrated in numerous studies, such as medical image classification and remote sensing [44–46]. In this work, the introduction of an attention mechanism aims to help the model select more relevant features from multi-omics data, thereby improving the accuracy of patient survival risk prediction. Specifically, for high-dimensional and complex multi-omics data, traditional convolutional neural networks may struggle to effectively capture key features. The channel attention mechanism enhances the model’s ability to focus on important features by adaptively adjusting the weights of different channels.

The channel attention mechanism utilizes Global Average Pooling and Global Max Pooling to assist the model in extracting key features from a global perspective:

Global Average Pooling aggregates the input features globally to extract the average information across the entire input, enabling the model to capture overall characteristics of the input features. Let the feature map be denoted as $\text{F}\in {\text{R}}^{\text{H}\times \text{W}\times \text{C}}$, where *H* is the height, W is the width, and C is the number of channels. The global average pooling for the C-th channel is given by:


$$\begin{array}{c} F_{avg}^{c}=\frac{\text{1}}{H\times W}\sum_{i=\text{1}}^{H}\sum_{j=\text{1}}^{W}F_{ij}^{c} \end{array}$$ (3)

Global Max Pooling, on the other hand, focuses on extracting the most prominent features, finding the regions that have the strongest response in a given channel. For the C-th channel, the global max pooling is as follows:


$$\begin{array}{c} F_{max}^{c}=\underset{i=\text{1},\ldots ,H;\;j=\text{1},\ldots ,W}{max}F_{ij}^{c} \end{array}$$ (4)

To process the global features obtained through average pooling and max pooling, fully connected layers with shared weights are used to reduce the number of parameters. The first fully connected layer uses the ReLU activation function to generate compressed features:


$$\begin{array}{c} M_{\text{1}}=ReLU\left(W_{\text{1}}F_{pool}+b_{\text{1}}\right) \end{array}$$ (5)

Where ${\text{W}}_{\text{1}}\in {\text{R}}^{\text{C}/\text{r}\times \text{C}}$ represents the weights of the first fully connected layer, $b_{\text{1}}$ is the bias term,  r is the reduction ratio.

The second fully connected layer projects the compressed features back to the original channel dimension, generating the attention weights for each channel:


$$\begin{array}{c} M_{\text{2}}=W_{\text{2}}M_{\text{1}}+b_{\text{2}} \end{array}$$ (6)

Where ${\text{W}}_{\text{1}}\in {\text{R}}^{\text{C}/\text{r}\times \text{C}}$ is the weight of the second fully connected layer, and $b_{\text{2}}$ is the bias term.

The channel attention mechanism assigns a weight to each channel, combining the results of Global Average Pooling and Global Max Pooling by addition to obtain the final weight matrix M:


$$\begin{array}{c} M=M_{avg}+M_{max} \end{array}$$ (7)

Finally, the model combines the input features with the attention weights through element-wise multiplication, enhancing the response of high-weight channels and suppressing low-weight channels:


$$\begin{array}{c} F^{\prime }=F⊙\sigma (M) \end{array}$$ (8)

### Selection of survival-related latent features

LASSO regression was applied to reduce dimensionality and select the most informative latent variables. This approach penalizes less relevant features and retains those with stronger associations to survival outcomes.

We applied the Cox proportional hazards (Cox-PH) model to evaluate the relationship between each latent feature and patient survival. Using the Python package lifelines (https://github.com/CamDavidsonPilon/lifelines), we constructed a univariate Cox-PH model and selected features with log-rank P-values (Wilcoxon test) < 0.05 as survival-associated variables.

Finally, the significant latent features extracted from each CA-CAE were combined into a new matrix for subsequent analysis.

### Cancer subtype classification

K-means clustering is used to assign samples with similar characteristics to different subgroups. This method randomly selects k centers initially, assigns each sample to the closest center based on the distance, recalculates the new centers after the assignment, and calculates the average distance of each cluster as the new centroid. This process is repeated until convergence, where cluster centers no longer change, and the total distance reaches a minimum value, which is then considered as the optimal value.

The K-means clustering objective function is as follows:


$$\begin{array}{c} J=\sum_{i=\text{1}}^{k}\underset{x_{j}\in C_{i}}{\sum \;}∥x_{j}-\mu_{i}{∥}^{\text{2}} \end{array}$$ (9)

Where J is the sum of squared distances from all samples to their respective cluster centers, $C_{i}$ is the i-th cluster, $x_{j}$ is the j-th sample, $\mu_{i}$ is the centroid of the i-th cluster, and $∥x_{j}-\mu_{i}{∥}^{\text{2}}$ represents the Euclidean distance between sample $x_{j}$ and centroid $\mu_{i}$.

To determine the optimal number of clusters, we set k = 2,3,4,5 and calculated the Silhouette coefficient and P−value for each value of k. Based on the Silhouette coefficient and P−value, we selected the appropriate number of clusters.

### Model evaluation metrics

#### Silhouette coefficient.

The silhouette coefficient is used to measure how similar each data point is to other data points within the same cluster, compared to data points in other clusters. It ranges between -1 and 1, where a higher value indicates that the data point is well matched to its own cluster and poorly matched to neighboring clusters. For the i-th data point, the silhouette coefficient $S_{i}$ is defined as follows:


$$\begin{array}{c} S_{i}=\frac{b_{i}-a_{i}}{\text{max}\left(a_{i},b_{i}\right)} \end{array}$$ (10)

Where $a_{i}$ represents the average distance between the i-th data point and all other points within the same cluster (intra-cluster distance), and $b_{i}$ represents the minimum average distance between the i-th data point and all points in any other cluster (nearest-cluster distance). The silhouette coefficient for all data points is averaged to represent the overall clustering quality. Silhouette coefficient values close to 1 indicate that the data is well clustered, while values close to -1 indicate poor clustering quality.

#### Log-rank test.

The log-rank test is used to compare the survival curves between different groups. We used the Kaplan-Meier estimator provided in the Python lifelines package to estimate survival curves and calculated the test statistic for differences between groups. The test statistic Z is given by:


$$\begin{array}{c} Z=\frac{\sum_{i=\text{1}}^{k}(O_{i}-E_{i})}{\sqrt{\sum_{i=\text{1}}^{k}V_{i}}} \end{array}$$ (11)

where $O_{i}$ is the observed number of events (e.g., deaths) in group i, $E_{i}$ is the expected number of events under the null hypothesis, and $V_{i}$ represents the variance of the number of events. A larger Z value indicates a greater difference between the groups. If the P-value of Z is less than 0.05, the difference is considered statistically significant.

#### Cox-PH concordance index.

The concordance index (C-index) is commonly used to evaluate the predictive accuracy of survival models, especially when the predicted output is time to an event. It measures how well the predicted survival times match the observed outcomes, and it is often used to assess the discriminative power of the Cox proportional hazards (Cox-PH) model. The formula for the C-index is as follows:


$$\begin{array}{c} C=\frac{\sum I\left(\hat{h}\left(X_{i}\right)>\hat{h}\left(X_{j}\right)\right)\cdot I\left(T_{i}<T_{j}\right)}{\sum I\left(T_{i}<T_{j}\right)} \end{array}$$ (12)

where $\hat{h}\left(X_{i}\right)$ represents the risk score of the i-th individual, $I(T_{i}<T_{j})$ is an indicator function that returns 1 if ${(T}_{i}<T_{j})$, and 0 otherwise. The C-index takes values between 0.5 and 1, where 0.5 indicates random predictions and values closer to 1 indicate better predictive accuracy.

#### Calinski–Harabasz (CH) index.

The Calinski–Harabasz index evaluates clustering quality by computing the ratio between the sum of between-cluster dispersion and within-cluster dispersion. A higher CH score indicates better-defined and more separated clusters.

#### Davies–Bouldin (DB) index.

The Davies–Bouldin index measures the average similarity between each cluster and its most similar one, using intra-cluster and inter-cluster distances. A lower DB index indicates more distinct and compact clusters.

#### Bayesian information criterion (BIC).

To evaluate model fit and complexity, the Bayesian Information Criterion was calculated using a Gaussian Mixture Model (GMM). A lower BIC value indicates a better balance between model accuracy and parsimony, helping to avoid overfitting during subtype determination.

#### Cross-validation and stability.

To comprehensively assess the robustness and reliability of the proposed CA-CAE model, multiple quantitative evaluation metrics were employed, including the Silhouette coefficient, Calinski-Harabasz (CH) index, Davies-Bouldin (DB) index, Bayesian Information Criterion (BIC), and Log-rank test. In addition, five-fold cross-validation and repeated clustering under different random seeds were conducted to evaluate model stability.

#### Chi-square test.

The chi-square test was used to determine whether there is a statistically significant association between two categorical variables. The test statistic is given by:


$$\begin{array}{c} \chi^{\text{2}}=\sum \frac{(O_{i}-E_{i}{)}^{\text{2}}}{E_{i}} \end{array}$$ (13)

The $\chi^{\text{2}}$ statistic is used to evaluate the degree of deviation between observed data and expected data. $O_{i}$ represents the observed value in category i, and $E_{i}$ represents the expected value in category i.

If the $\chi^{\text{2}}$ statistic is large, it indicates a significant difference between the observed and expected values, suggesting a potential association between the variables. The P-value reflects the significance of the test result. If P< 0.05, it is considered that there is a significant association between the two variables.

#### Interpretation of P-value and confidence interval.

The p-value obtained from the log-rank test measures whether the survival distributions between identified subtypes are significantly different. A smaller p-value indicates stronger evidence that the model successfully distinguishes prognostically distinct patient subgroups.

The concordance index (C-index) and its 95% confidence interval (CI) were calculated through bootstrapping to evaluate the reliability of the survival prediction. Narrow CIs indicate higher estimation stability and model robustness.

### Model performance comparison

ProgCAE achieved better survival risk prediction performance compared to other methods, demonstrating its effectiveness on multiple cancer datasets. Specifically, ProgCAE showed superior prediction accuracy and stability compared to PCA, highlighting its enhanced feature extraction capabilities and better suitability for survival analysis.

DeepProg, a deep feature extraction model, was also evaluated for comparison. We performed Cox-PH analysis on the extracted features from each model and conducted survival analysis to assess the consistency of predictions with the actual outcomes.

We compared the performance of DeepProg, ProgCAE, PCA, CA-CAE and NMF on several datasets. We used the SVM classifier implemented in the Python scikit-learn package to classify the features. For fairness, all baseline models followed the same downstream framework as CA-CAE. In the PCA and NMF baselines, the dimensionality-reduced features obtained from each method were further analyzed using the same LASSO + Cox proportional hazards pipeline for feature selection and survival analysis.Thus, PCA and NMF only replaced the feature extraction component, while the rest of the analysis procedure remained identical. Other deep learning baselines were also reproduced using the same data preprocessing, cross-validation, and evaluation metrics to ensure comparability. For parameter tuning, we used 5-fold cross-validation with a grid search to determine the best model parameters. The model with the highest C-index was selected for further survival analysis across 15 cancer types.

### Ablation experiments

To evaluate the contribution of individual components in the CA-CAE framework, we performed ablation experiments on the LUAD multi-omics dataset. Starting from the full CA-CAE pipeline, we constructed two reduced variants: (i) a model without the channel attention module and (ii) a model without the Cox-based prognostic feature filtering. All variants were trained with the same input features, preprocessing steps, and training hyperparameters as the full model to ensure comparability. The purpose of these ablations was to determine whether attention-guided feature refinement and survival-driven feature selection are necessary steps for building a stable subtype-identification pipeline.

### Building SVM

Support Vector Machine (SVM) is a useful supervised machine learning classifier. Labels obtained through K-means clustering were used to construct SVM classifiers for the ACC, BLCA, and LUAD datasets. We first extracted the top 50 features with the highest variance for each omics type using the previous normalization, and combined them to form a new training matrix Z. The data was split into training and testing sets in a 7:3 ratio. The training set was used to train the model, while the testing set was used to evaluate predictive performance.

To find the optimal hyperparameters for the classifier, we used the SVM algorithm implemented in the scikit-learn package to perform a grid search on Z with 5-fold cross-validation.

### Differential gene analysis and survival analysis of multi-omics data

Differential gene expression analysis was performed on the LUAD dataset to identify differentially expressed genes across various cancer subtypes. We extracted 20 genes as candidate markers for further study and assessed their association with patient survival. The results showed significant differences between cancer subtypes, indicating that these genes may play a key role in the progression of LUAD.

### Functional enrichment analysis

Survival-associated genes obtained from the CA-CAE pipeline were analyzed for Gene Ontology (GO) and KEGG pathway enrichment using the R package clusterProfiler. This analysis was performed to explore whether the prognostic genes were concentrated in immune-related or tumor progression–related biological processes and signaling pathways.

### Relationship between clinical traits and cancer subtypes

To further analyze the LUAD dataset, we performed chi-square tests between clinical traits (Stage, T (tumor classification), N (lymph node status), and M (metastasis)) and cancer subtypes. The results showed significant associations between clinical traits and subtypes. For instance, Stage and N classification were significantly associated with subtypes (p-value < 0.05), suggesting that these clinical factors are closely related to cancer subtype classification and may serve as valuable prognostic indicators.

### Association analysis of CA-CAE subtypes with WGD and NMF-based subtypes

To assess whether the molecular subtypes identified by CA-CAE capture biologically meaningful variation, we tested their associations with whole-genome doubling (WGD) status and with subtypes obtained from a previously reported NMF-based clustering workflow. For WGD, we first assigned each sample to a CA-CAE subtype and then cross-tabulated subtype labels with binary WGD calls. We used χ^2^ tests to evaluate dependence between subtype and WGD status and calculated effect size (Cramér’s V) to quantify the strength of association. For the comparison with NMF, we aligned sample IDs between the two clustering results and computed Adjusted Rand Index (ARI) and Normalized Mutual Information (NMI) to measure label-level agreement. This analysis was designed to test subtype plausibility and concordance with an established omics-based clustering strategy, not to reproduce its exact partition.

### External validation on CPTAC proteogenomic datasets

To examine the transferability of CA-CAE to other multi-omics settings, we applied the same preprocessing and modeling steps to proteogenomic cohorts from the Clinical Proteomic Tumor Analysis Consortium (CPTAC). Proteomic and transcriptomic profiles were first matched at the patient level, and only samples with complete survival information and all required omics layers were retained. Each omics layer then underwent the same normalization, feature reordering, and attention-enhanced CAE encoding as in the TCGA experiments. Survival-related latent features were selected with the LASSO+Cox procedure, and clustering was carried out on the integrated feature space. This external test was intended to check whether the model can operate on heterogeneous multi-omics combinations collected in different projects and still produce survival-oriented patient groupings.

## Supporting information

S1 Table Clustering evaluation metrics for cancer subtypes. (DOCX)

S2 Table Sensitivity analysis of model performance under different feature selection thresholds. (DOCX)
