# Supplementary material for: CA-CAE: A deep learning-based multi-omics model for pan-cancer subtype classification and prognosis prediction
Source: PLoS Comput Biol. 2026 Feb 20;22(2):e1014015. doi: 10.1371/journal.pcbi.1014015 (PMC12948314; doi:10.1371/journal.pcbi.1014015)
Supplement: S1 Table — (DOCX) [file pcbi.1014015.s001.docx]

**S1 Table** Clustering Evaluation Metrics for Cancer Subtypes

| Cancer | Silhouette | CH | DB | BIC | p-value |
| --- | --- | --- | --- | --- | --- |
| ACC | 0.32 | 68.33 | 1.08 | -5750.74 | 2.10E-05 |
| BLCA | 0.34 | 299.66 | 1.11 | -4855.40 | 1.30E-04 |
| CESC | 0.24 | 102.89 | 1.53 | 9567.67 | 1.20E-04 |
| CHOL | 0.70 | 153.33 | 0.40 | 151.40 | 8.60E-03 |
| COAD | 0.49 | 396.07 | 0.75 | 1153.52 | 9.10E-04 |
| KICH | 0.14 | 12.70 | 2.08 | 1542.09 | 3.10E-04 |
| LAML | 0.22 | 136.03 | 1.46 | -2471.27 | 4.80E-03 |
| LUAD | 0.43 | 372.17 | 0.85 | 820.04 | 8.40E-07 |
| LUSC | 0.25 | 43.86 | 1.33 | -4107.04 | 5.30E-03 |
| MESO | 0.23 | 89.49 | 1.59 | 13503.41 | 2.60E-05 |
| SARC | 0.43 | 381.48 | 0.86 | -29458.86 | 2.50E-04 |
| STAD | 0.31 | 267.81 | 1.28 | 7410.22 | 6.90E-04 |
| THCA | 0.33 | 259.44 | 1.18 | -22529.55 | 5.80E-04 |
| UCEC | 0.37 | 61.36 | 1.09 | -5215.41 | 7.60E-05 |

In order to identify the best clustering solution for each cancer type, we used the following criteria based on standard clustering evaluation metrics:

Silhouette Score: The higher the silhouette score, the better the clustering. A higher score indicates that the data points are well-clustered and distinct from other clusters.

Calinski-Harabasz (CH) Index: A higher CH score signifies better-defined clusters with larger between-cluster variance and smaller within-cluster variance.

Davies-Bouldin (DB) Index: A lower DB score indicates better separation between clusters, meaning that the clusters are well-separated and compact.

Bayesian Information Criterion (BIC): A lower BIC score suggests a more appropriate number of clusters with a better model fit, penalizing complexity.

p-value: A smaller p-value (typically < 0.05) indicates statistically significant clustering results.

For each cancer type, we selected the clustering configuration that maximized the silhouette score, maximized the CH index, minimized the DB index, minimized the BIC score, and yielded the most significant p-value. These criteria were used to ensure the best possible clustering performance for each cancer dataset.
