# Supplementary material for: CA-CAE: A deep learning-based multi-omics model for pan-cancer subtype classification and prognosis prediction
Source: PLoS Comput Biol. 2026 Feb 20;22(2):e1014015. doi: 10.1371/journal.pcbi.1014015 (PMC12948314; doi:10.1371/journal.pcbi.1014015)
Supplement: S2 Table — (DOCX) [file pcbi.1014015.s002.docx]

**S2 Table**  Sensitivity analysis of model performance under different feature selection thresholds.

| Feature Counts (RNA/miRNA/Meth) | C-index | P-value |
| --- | --- | --- |
| 1000/50/500 | 0.582 | 1.5E−04 |
| 2700/90/900 | 0.639 | 1.2E−06 |
| 3000/100/1,000 | 0.643 | 8.4E−07 |
| 3300/110/1,100 | 0.644 | 7.9E−07 |
| 5000/200/1500 | 0.648 | 6.5E−07 |

To determine the optimal input dimensions for the CA-CAE model, we conducted a sensitivity analysis by varying the number of selected high-variance features. As presented in Table S2, reducing the feature counts to a lower threshold (1000mRNA, 50miRNA, 500Meth) resulted in a significant performance drop, with the C-index decreasing to 0.582 , indicating an excessive loss of prognostic information.Conversely, increasing the feature counts to a high-dimensional setting (5000mRNA, 200miRNA, 1500Meth) yielded a C-index of 0.648. Compared to our selected baseline (C-index = 0.643), this represents a marginal improvement of only 0.78%, yet it incurs substantially higher computational costs. Furthermore, fluctuations within the ±10% range of the baseline (C-index 0.639-0.644) were negligible. Therefore, the threshold of 3000 mRNA, 100miRNA, and 1,000 Methylation features was selected to achieve the optimal balance between predictive accuracy and model efficiency.
